# The Effects of a Digital Well-being Intervention on Older Adults: Retrospective Analysis of Real-world User Data

**DOI:** 10.2196/39851

**Published:** 2022-09-02

**Authors:** Eliane Boucher, Ryan Honomichl, Haley Ward, Tyler Powell, Sarah Elizabeth Stoeckl, Acacia Parks

**Affiliations:** 1 Twill New York, NY United States

**Keywords:** mobile apps, mental health, older adults, technology adoption, digital health, mobile phone

## Abstract

**Background:**

Digital interventions have been shown to be effective for a variety of mental health disorders and problems. However, few studies have examined the effects of digital interventions in older adults; therefore, little is known about how older adults engage with or benefit from these interventions. Given that adoption rates for technology among people aged ≥65 years remain substantially lower than in the general population and that approximately 20% of older adults are affected by mental health disorders, research exploring whether older adults will use and benefit from digital interventions is needed.

**Objective:**

This study aimed to examine the extent to which older adults engaged with a digital well-being intervention (Happify) and whether engaging with this program led to improvements in both subjective well-being and anxiety symptoms.

**Methods:**

In this retrospective analysis, we analyzed data from 375 real-world Happify users aged ≥65 years who signed up for the platform between January 1, 2019, and December 23, 2021. Changes in well-being and anxiety symptoms across 42 to 182 days were assessed using responses to the in-app assessment, which users were prompted to take every 2 weeks, and were compared among users who engaged with the program at the recommended level (ie, 2 or more activities per week) or below the recommended level.

**Results:**

In all, 30% (113/375) of the sample engaged with the platform at the recommended level (ie, completed an average of 2 or more activities per week), and overall, users completed an average of 43.35 (SD 87.80) activities, ranging from 1 to 786, between their first and last assessment. Users were also active on the platform for an average of 19.36 (SD 27.16) days, ranging from 1 to 152 days. Moreover, older adults who engaged at the recommended level experienced significantly greater improvements in subjective well-being (*P*=.002) and anxiety symptoms (*P*<.001) relative to those who completed fewer activities.

**Conclusions:**

These data provide preliminary evidence that older adults engage with and benefit from digital well-being interventions. We believe that these findings highlight the importance of considering older adult populations in digital health research. More research is needed to understand potential barriers to using digital interventions among older adults and whether digital interventions should be modified to account for this population’s particular needs (eg, ensuring that the intervention is accessible using a variety of devices). However, these results are an important step in demonstrating the feasibility of such interventions in a population that is assumed to be less inclined toward digital approaches.

## Introduction

### Background

According to the United Nations, the proportion of the global population >65 years is increasing and is expected to reach 1 in 6 people by 2050, compared with 1 in 11 in 2019 [1]. In the United States, the US Census Bureau estimated that by 2030, the population of older adults will exceed that of children for the first time, with 1 in 5 Americans being of retirement age [2]. Although the aging population has spurred discussions surrounding the added burden of chronic illness and complex medical conditions in this cohort [3-6], comparatively less attention has been dedicated to understanding the implications of poor mental health in this “silver tsunami.”

The World Health Organization reports that 1 in 5 adults aged ≥60 years is affected by a mental or neurological disorder, excluding headaches, and these disorders account for 17.4% of the years lived with disability [7]. The most common mental health disorders in this age group are depression and anxiety, affecting approximately 7% and 3.8% of older adults worldwide, respectively [7]. In the United States and Australia, the prevalence of depressive symptoms alone among older adults is estimated to be 9.8% [8]. Poor mental health in older adults subsequently contributes to elevated health care costs [9], directly and indirectly by worsening comorbid health conditions [10]. Moreover, research suggests that approximately 1 in 4 older adults with mental health disorders report experiencing discrimination, including racism and ageism, compared with 1 in 10 older adults without mental health disorders. Among those with mental health disorders, this discrimination was also more likely to occur within health care settings, thus increasing the likelihood that the individual will delay treatment or not seek treatment at all [11].

In fact, older adults are less likely than middle-aged adults to seek mental health care [12]. In a study, 6.5% of older adults self-reported some level of mental health care in the previous 12 months, but 65.9% of respondents with clinical levels of depression and 72.5% of those with anxiety never received treatment [13]. Research suggests that the lack of treatment seeking may not be related to perceptions of mental health care but to access. Specifically, a study found that older adults were more likely to indicate that access to mental health care was important but less likely to indicate that they had access to such care [14]. Although lower rates of treatment seeking may be because older adults are less likely to report having insurance coverage for mental health services compared with younger adults [14], it is further compounded by the shortage of mental health professionals specializing in geriatric populations [15].

Given these difficulties in accessing mental health care, the need to explore scalable and affordable options for mental health care is imperative as a growing proportion of the population enters old age and requires more services. Over the past 2 decades, a number of digital interventions have been developed to address the general unmet need for mental health care [16], and research suggests that these can effectively help improve mental health, including depression, anxiety, and stress [17-20]. However, research on whether older adults will engage with or benefit from digital interventions is limited.

We should be careful not to assume that the evidence suggesting that digital interventions are usable and effective within the general population applies to older adults. Although an increasing number of older adults reports owning a smartphone and using the internet, the proportion of older adults owning smartphones or having access to broadband services at home is still lagging behind that of younger age groups. For example, a national survey of adults in the United States conducted by the Pew Research Center in 2021 showed that 85% of respondents indicated owning a smartphone, whereas among older adults, only 61% reported owning smartphones. Among those aged ≥75 years, only 43% owned smartphones [21]. Age-related issues with manual dexterity and vision as well as a lack of confidence in using new technologies may contribute to the slower adoption of digital interventions and tools among older adults [22].

However, the few studies conducted with older adults suggest that those who engage with digital interventions show improvements in mental health outcomes. A meta-analysis of 9 studies exploring the effects of internet-based cognitive behavioral therapy (CBT) in older adults (mean age 66 years) found that these programs were generally effective at reducing depressive symptoms, although there was some evidence that their effectiveness was negatively related to the user’s age [23]. However, limited research on the impact of digital interventions on loneliness has shown no significant improvement in loneliness among older adults [24]. Other research suggests that engagement with digital interventions may improve with age [25-27]. One study of patients prescribed internet-based CBT in Australia found that patients aged ≥60 years were more likely to complete all treatment modules than younger patients, and improvements in psychological distress and disability were consistent across age groups [25]. However, researchers have argued that these studies provide little information about the uptake and engagement of digital interventions among older adults outside of controlled research conditions [28].

### Objectives

The purpose of this study was to explore whether older adults engaged with a publicly available digital intervention, the *Happify* wellness program, and the extent to which engaging with this intervention led to improvements in mental health over time. Happify is a self-guided wellness program that aims to improve psychological well-being by delivering brief gamified activities adapted from evidence-based activities from various therapeutic approaches. Previous research has shown that completing 2 or more activities via Happify per week led to significant improvements in subjective well-being and anxiety symptoms after 6 weeks [29-31], but none of these studies examined the effects of age or focused specifically on older adults. Therefore, in the current retrospective analysis, we analyzed data from real-world Happify users who self-reported being aged ≥65 years to determine whether completion of intervention activities was related to changes in subjective well-being and anxiety symptoms after at least six weeks of use.

## Methods

### Study Design

This study was a retrospective analysis of real-world Happify users who signed up for the program between January 1, 2019, and December 23, 2021.

### Sample Selection

When signing up, all users were prompted to complete an onboarding questionnaire after downloading the Happify application or accessing the website. This questionnaire inquired about demographic information, including age group, gender, relationship status, and employment status, intended to help tailor the program for individual users. Upon completing this questionnaire, users must agree to the terms of the service and privacy policy before creating their account, which includes language indicating that their data may be used for research purposes. All data presented here were generated by real-world users as part of the standard user experience and stored on secure company servers, and only deidentified data were extracted for analysis.

Data from all users located in the United States who selected “65 or older” as their age category when responding to the onboarding questionnaire and who completed at least two in-app assessments were initially considered. To be included in the analysis, users also had to meet the following criteria: (1) complete at least two in-app assessments within 182 days, (2) the time between their first and last assessments was no less than 42 days, and (3) complete at least one Happify activity between their first and last assessments.

### Ethics Approval

The use of Happify consumer data for retrospective analyses, such as this one, where data represent only those of users who naturally sign up for Happify and engage with the generic version of the program (ie, where no content or assessments have been changed for the purposes of research), was reviewed by IntegReview, an independent institutional review board, and labeled as exempt research (HLS-018).

### Materials and Procedure

#### Digital Well-being Intervention

**Figure 1:**
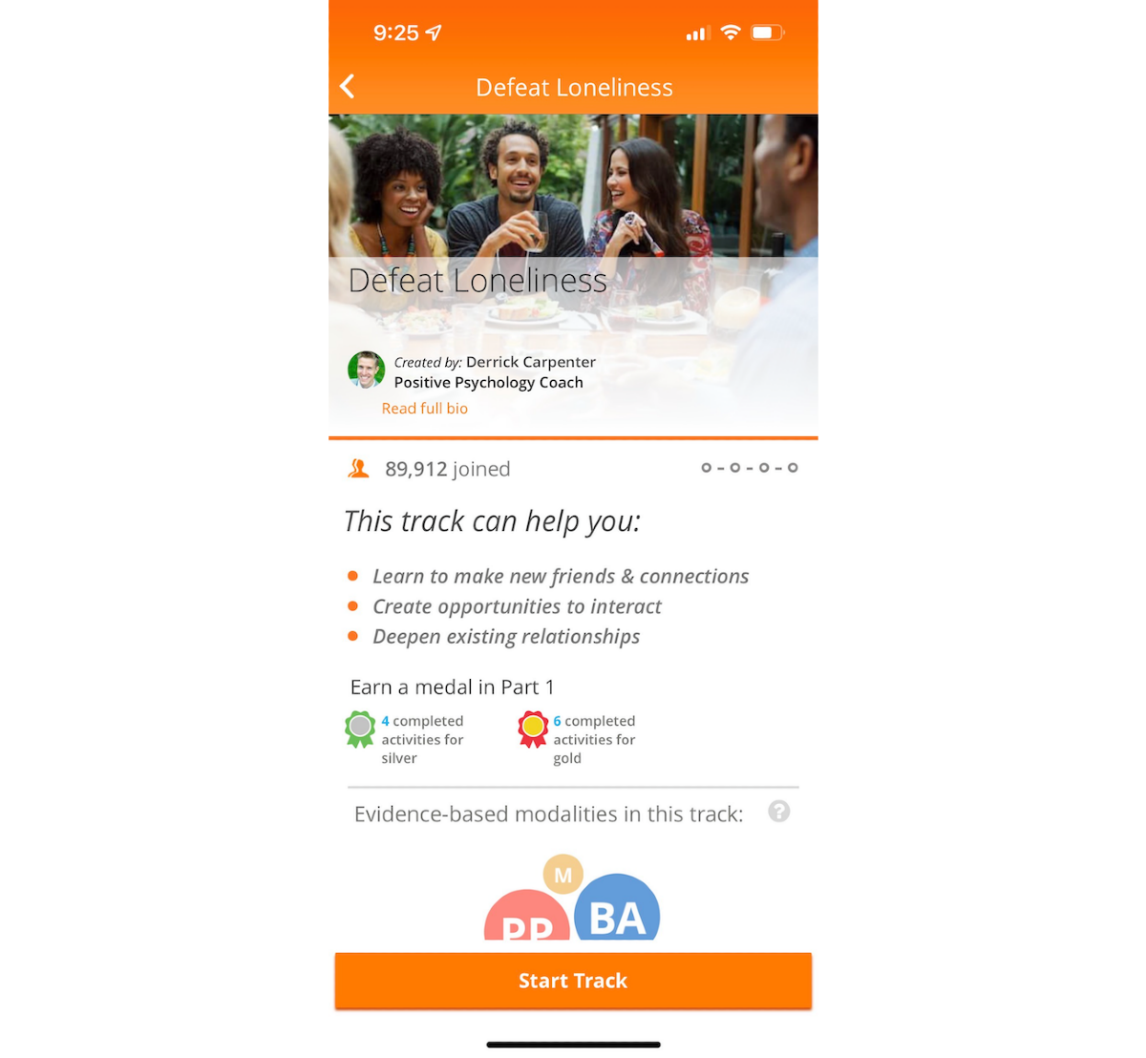
Screenshot of a Happify track on the smartphone app version.

**Figure 2:**
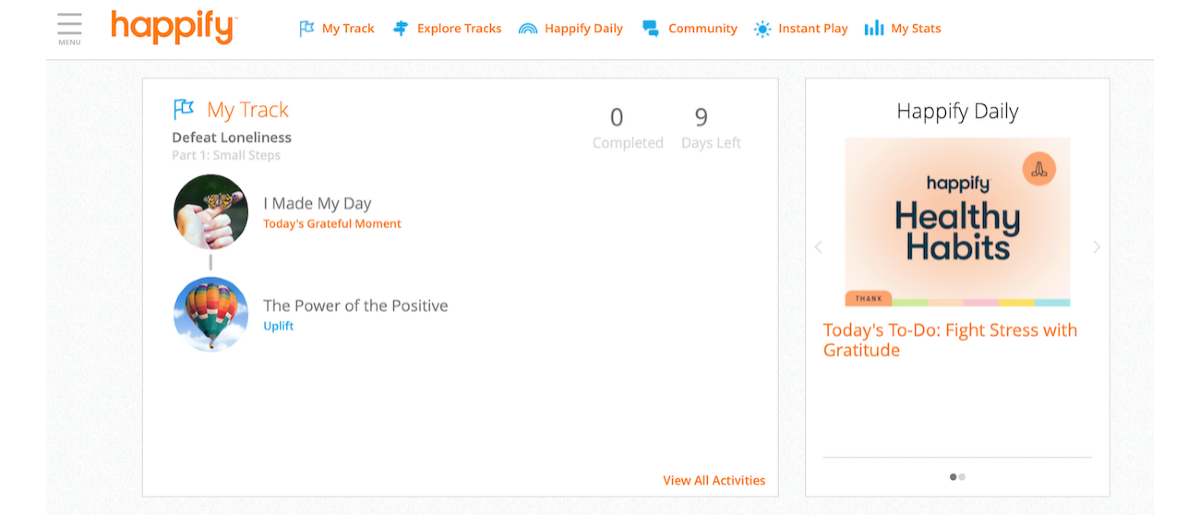
Screenshot of Happify track part on the web-based version.

Happify is a self-guided wellness program that draws on various theoretical approaches to improve well-being including CBT [32], mindfulness-based stress reduction [33], positive psychology [34], acceptance and commitment therapy [35], and behavioral activation [36]. Activities based on these therapeutic approaches were developed by identifying activities within each evidence-based approach (ie, demonstrated effectiveness in at least two different studies and with different samples) [37]. These activities are then organized into “tracks,” which are meant to help users address a specific area of concern, such as coping with stress or improving sleep (Figure 1). Each track consists of 4 parts, and users progress through the track by completing a percentage of the activities within each part (Figure 2). Users can change tracks at any time, and they can also access activities outside tracks via the instant play feature. A more detailed description of the Happify program is available in a previous research [31].

#### Assessment

As part of the regular Happify program, users are prompted to complete an in-app assessment the second time they log into the platform and every 2 weeks thereafter. This assessment consists of two measures: a proprietary measure of subjective well-being, the Happify Scale, and the Generalized Anxiety Disorder 2 (GAD-2) scale [38].

The in-app assessment is optional; therefore, users may choose to skip or delay the assessment when prompted. Owing to the optional nature of the assessment, many users do not provide this outcome data. Among those who completed the assessments, the time between assessments and the number of assessments also varied across users. Thus, the time between assessments was treated continuously in our analysis. For the analysis, assessments completed within 182 days after the first assessment were included. Consequently, the potential range of time between a user’s first and last assessment was 42 to 182 days.

#### Happify Scale

The Happify Scale is a 9-item, proprietary measure of subjective well-being. This scale was designed to measure two primary components of subjective well-being: positive emotionality and life satisfaction [31]. A total of 4 items assessed the frequency of positive emotions over the past month (eg, “In the past month, how often have you felt joyous, exuberant, inspired, and/or awestruck?”) and were based on the Positive and Negative Affect Schedule, a widely used measure of positive and negative emotions [39]. These items are rated on a 5-point scale ranging from 0 (*never*) to 4 (*very often [almost every day]*). The 5 remaining items assess user satisfaction across various life domains (eg, “How satisfied do you feel with the relationships in your life?”) and were based on the Satisfaction with Life Scale [40], which is a widely used measure of life satisfaction. These items were rated on a 7-point scale ranging from 0 (*very dissatisfied*) to 6 (*very satisfied*). Scores on each subscale were computed by summing the ratings and computing a percentage score ranging from 0 to 100. A composite score was then generated by averaging the 2 percentages, with higher scores indicating greater subjective well-being.

As described elsewhere [29], an unpublished internal validation study was conducted on the Happify Scale with 559 adults recruited from the general population using Amazon MTurk. In this study, the Happify Scale was shown to have good internal consistency (α=.89), and the internal consistency for each subscale was at least adequate (positive emotions: α=.72; life satisfaction: α=.88). The subscales were also strongly associated with the scales they were based on (*r*=0.76-0.80), and the total Happiness Scale score was strongly correlated with the Subjective Happiness Scale (*r*=0.78) and the Center for Epidemiological Studies Depression Scale (*r*=−0.75), in the predicted direction.

#### GAD-2 Scale

The GAD-2 [38] is a 2-item screening tool for generalized anxiety disorder, consisting of the first 2 items from the longer 7-item GAD scale. Respondents indicate the extent to which they have been bothered by each of the issues over the past 2 weeks on a scale from 0 (*not at all*) to 3 (*nearly every day*). The ratings were summed so that higher scores indicated greater anxiety symptoms. Although the GAD-2 is typically used as a screening tool, where a score of ≥3 indicates the likelihood of an anxiety disorder, it has also been used as a continuous scale to assess changes in anxiety in response to treatment [41].

### Statistical Analysis

To examine changes in well-being and anxiety symptoms, we fit linear mixed effects models for each outcome, treating days from the first assessment to each subsequent assessment as a fixed effect. We selected this approach because of its ability to handle a varied number of assessments across participants at various time points. Models were fitted with R (version 4.1.0 [42]), using the nlme package [43]. Models with random intercepts only and those with both random intercepts and random slopes were evaluated using the Akaike Information Criterion. The final models were fitted using random intercepts. Owing to the variability in the number of assessments and time between assessments, we fitted a continuous autoregressive error structure, conditional autoregressive (1) [44]. For each outcome, we fit a main effects model and then a model with an added interaction between time and use level (recommended vs below recommended). Each model included time, use (recommended level vs below recommended level), gender (dummy coded as “woman”), relationship status (dummy coded as “in a relationship”), number of chronic conditions, number of activities completed before the first assessment, and initial scores on the other outcome variables as predictors. Model comparison and selection were then made using Akaike Information Criterion; the interaction model better fit the data for both well-being and anxiety symptoms and thus, are reported here. All statistical tests were 2-tailed with an α criterion of .050.

## Results

### Sample and Demographics

During the qualifying period, 1292 new users reported being ≥65 years, residing in the United States, and completing at least two in-app assessments. After excluding participants whose second assessment was >182 days from their first assessment (n=89), whose last assessment was <42 days from their first (n=476), who completed no activities between their first and last assessments (n=306), or who had missing demographic information (n=46), our final sample consisted of 375 older adults. The sample demographics are presented in Table 1.

Users completed an average of 4.17 (SD 2.63) assessments, ranging from 2 to 13 assessments, with an average of 49.89 (SD 38.52) days between assessments. Initial well-being was not correlated with the number of completed assessments (*r*=.06) or with the average number of days between assessments (*r*=−.07), whereas initial anxiety symptoms were significantly correlated with both (r=−0.17 and 0.21, respectively; *P*<.001).

**Table 1:** Baseline sample characteristics (N=375).

Characteristic			Value
**Gender^a^, n (%)**			
	Woman	297 (79.2)	
	Man	76 (20)	
	Other	2 (1)	
**Relationship status, n (%)**			
	In a relationship	265 (70.7)	
	Single	110 (29)	
**Chronic conditions**			
	Users with at least one chronic condition, n (%)	269 (71.7)	
	Number of chronic conditions, mean (SD)	1.39 (1)	
**Self-reported chronic physical conditions, n (%)**			
	Arthritis	56 (15)	
	Asthma	17 (5)	
	Cancer	12 (3)	
	Chronic pain	70 (19)	
	Diabetes	37 (10)	
	Eczema	8 (2)	
	Heart disease	4 (1)	
	High blood pressure and/or cholesterol	127 (33.9)	
	Insomnia	80 (21)	
	Migraine	19 (5)	
	Multiple sclerosis	2 (1)	
	Psoriasis	4 (1)	
	Rheumatoid arthritis	10 (3)	
	Other conditions	76 (20)	

^a^During onboarding, users are asked “Everyone’s Different: Tell Us Your Gender.” Before October 2020, response options were “male,” “female,” and “none of the above”; after this time, response options were changed to “man,” “woman,” and “none of the above.” Users who selected “male” or “man” are both represented in this table as “man,” whereas those who selected “female” or “woman” are both represented under “woman.”

### Use

We were able to verify how 326 of the 375 users (86.9%) accessed the Happify program. A small proportion of these users (54/326, 16.6%) accessed the program exclusively using a computer. Older adults were more likely to access Happify either using a smartphone or tablet (129/326, 34.3%) or a mix of devices (143/326, 43.8%). Among those who used either a smartphone or a tablet, the program was accessed primarily via a smartphone (117/129, 90.5%) compared with the tablet (12/129, 9.5%). Among those who used all 3 devices to access the program, access via a smartphone was the most common (mean 50.78%, SD 22.46%; range 0% -75%), followed by access via a computer (mean 36.12%, SD 10.63%; range 20%-50%) and a tablet (mean 13.10%, SD 18.25%; range 0%-25%).

The sample use statistics are listed in Table 2. Overall, older adult users completed an average of 43.35 (SD 87.80) activities, ranging from 1 to 876 activities, between their first and last assessment. On average, more activities were completed within a dedicated track (mean 35.47, SD 67.31; range 0-415) compared with activities completed via the instant play feature (mean 7.88, SD 37.56; range 0-558). A total of 113 (30.1%) of the 375 retained users engaged with the program at the recommended level of 2 activities per week during that period, which is consistent with other Happify research with a different sample [31]. The older adults in our sample also had an average of 19.36 (SD 27.16) active days on Happify, ranging from 1 to 152 days between their first and last assessment.

**Table 2:** Characteristics of engagement with Happify program.

		Value, mean (SD)	Value, range
Number of in-app assessments		4.17 (2.63)	2-13
Days between first and last assessments		104.54 (46.55)	42-182
Number of days between assessments		49.89 (38.52)	13.75-177
Activities completed before first assessment		1.22 (1.75)	0-19
**Activity between first and last assessment**			
	Total activities completed	43.35 (87.80)	1-786
	Track activities completed	35.47 (67.31)	0-415
	Instant play activities completed	7.88 (37.56)	0-588
	Days between first and last activity	92 (63.20)	1-152
	Active days^a^	19.36 (27.16)	1-152

^a^Any day when a user logged on to the Happify platform and completed an activity but does not include days when the user may have logged on without completing an activity (including completing the assessment).

### Subjective Well-being

Across the sample, the mean well-being score on the first assessment was 52.56 (SD 19.82), ranging from 5 to 99. This is below the 50th percentile of the Happify Scale in the general population (ie, a score of 61-63) [29]. We found that older adults with a higher number of chronic conditions had lower Happify Scale scores overall, B=−1.58 (95% CI −2.72 to −0.45; *P*=.007), which is consistent with research showing that health status is correlated with subjective well-being [45]. Not surprisingly, older adults with higher levels of anxiety symptoms on their first assessment also had lower levels of subjective well-being overall, B=−5.24 (95% CI −6.10 to −4.38; *P*<.001). These effects were consistent for both the main effects and the interaction models.

We also found significant main effects for both use (B=4.38; 95% CI 1.00-7.77; *P*=.011) and time (B=0.03; 95% CI 0.01-0.04; *P*<.001). However, these effects were qualified by a significant time×use interaction when added to the model (B=0.04; 95% CI 0.02-0.07; *P*=.002), and the main effects were no longer significant for use (B=−1.884; 95% CI −1.874 to 5.641; *P*=.33) or time (B=−0.012; 95% CI −0.005 to 0.029; *P*=.18).

**Figure 3:**
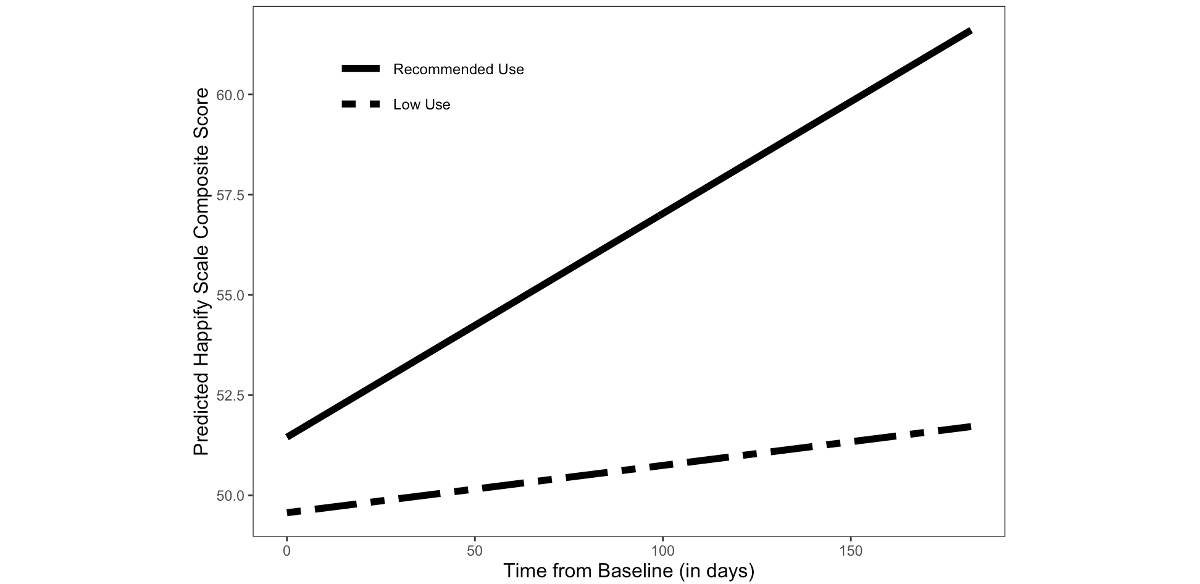
Changes in subjective well-being (as measured by the Happify Scale) over time as moderated by use (recommended use: average of ≥2 activities per week; low use: average of <2 activities per week).

As shown in Figure 3, older adults who completed an average of 2 or more activities per week while on Happify reported significantly greater improvements in subjective well-being than did those who completed fewer activities. More specifically, those who completed an average of 2 or more activities per week had an average improvement of 24.5% (SD 79.3%) in their Happify Scale scores compared with 11.7% (SD 45.5%) among those who engaged below the recommended level. No other effects were significant in either model.

### Anxiety

The mean GAD-2 scores on the first assessment were 2.14 (SD 1.82), ranging from 0 to 6, and 116 of the 375 (30.9%) users scored above the cutoff (ie, scores between 3 and 6) for likely anxiety disorder. In addition to the significant main effects of use (B=−0.31, 95% CI −0.60 to −0.02; *P*=.03) and time (B=−0.002, 95% CI: −0.004 to −0.001; *P*<.001), the only other significant main effect was for initial Happify Scale scores. Older adults with higher Happify Scale scores on their first assessment also had significantly lower levels of anxiety symptoms overall (B=−0.04, 95% CI −0.05 to −0.04; *P*<.001).

**Figure 4:**
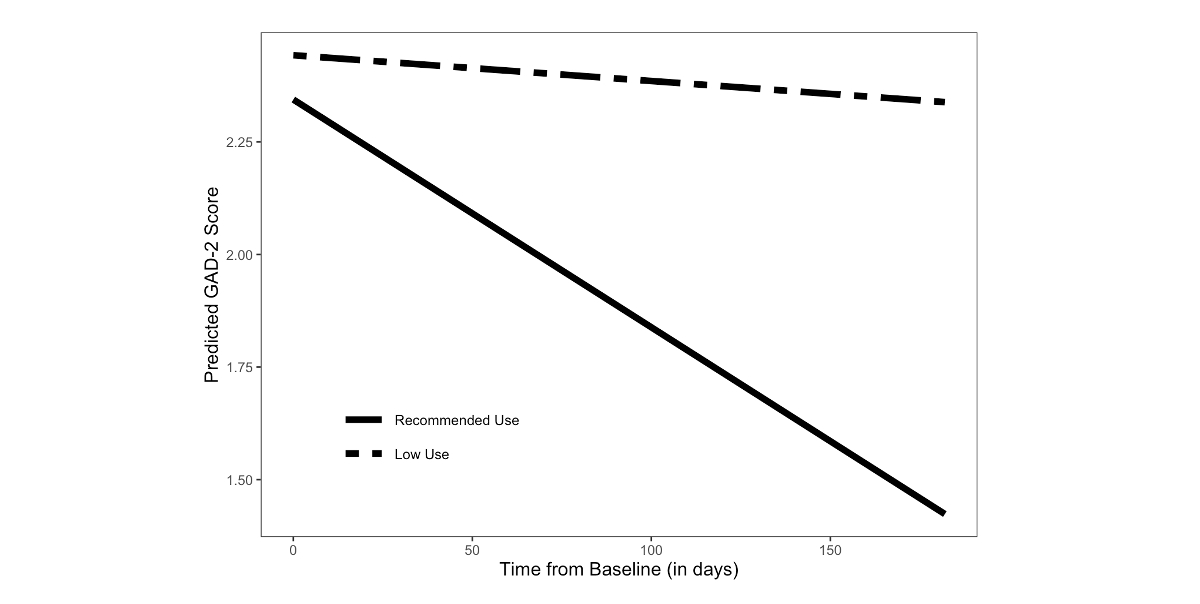
Changes in anxiety symptoms (as measured by the Generalized Anxiety Disorder 2 [GAD-2] scale) over time as moderated by use (recommended use: average of ≥2 activities per week; low use: average of <2 activities per week).

As with subjective well-being, both main effects for use and time were qualified by a significant use×time interaction (B=−0.004, 95% CI −0.007 to −0.002; *P*<.001) and were no longer significant once this interaction was added to the model (use (B=−0.098, 95% CI −0.409 to 0.212; *P*=.54); time (B=−0.001, 95% CI −0.002 to 0.001; *P*=.05). As depicted in Figure 4, older adults who completed an average of 2 or more activities per week reported significantly greater improvements in anxiety symptoms than those who completed fewer activities. More specifically, among older adults who engaged with the program at the recommended level, there was a 25.6% (SD 58.3%) improvement in GAD-2 scores compared with 10.5% (SD 88.3%) improvement among those who engaged below the recommended level.

## Discussion

### Principal Findings

The purpose of this analysis was to explore whether older adults would engage with a digital well-being intervention and the extent to which engaging with this intervention led to improvements in subjective well-being and anxiety symptoms. Our results show that approximately one-third of older adults who qualified for our analysis engaged with the program at the recommended level, which is consistent with the rates of engagement reported in other analyses of Happify users that did not include older adults [30].

Our results further showed that when older adults engaged at this optimal level, they experienced significant improvements in both subjective well-being and anxiety symptoms over time. These improvements are also comparable (or better) to those reported in other populations. For instance, a real-world analysis of Happify users found a 10.47% improvement in positive emotions (a subscale from the Happify Scale) over 8 weeks [31], whereas a more recent analysis of Happify users with self-reported migraines, where <1% of the sample was represented by older adults, found an average improvement of 23.5% in subjective well-being and 26.05% in anxiety symptoms [46]. The magnitude of change observed in the current analysis is consistent with those observed in the migraine analysis and better than what was reported in the study by Carpenter et al [31], with improvements in well-being averaging 24.5% and improvements in anxiety symptoms averaging 25.6%. These findings add to the growing body of literature suggesting that although technology adoption remains lower among older adults [21], they nevertheless respond to digital interventions [23,25-27,47]. By examining data from real-world users, our data provide evidence that even outside controlled research environments, older adults will sign up for digital well-being interventions and engage with them at comparable rates to younger users. This is important to understanding whether digital interventions are a viable means of addressing the unmet need for mental health care among older adults, regardless of their efficacy. If older adults do not engage with digital interventions, they cannot improve access to care.

### Improving Uptake of Digital Interventions Among Older Adults

Our data show that older adults can benefit from digital well-being interventions when they engage with them and that those who adopt this technology appear to engage at rates similar to what we find in younger age groups. However, it is worth noting that the uptake of these digital interventions was lower than that in other age groups. In the case of the Happify program, older adults who qualified for our analysis made up <3% of the general user base that met all other criteria for inclusion. Therefore, it is important to explore methods to increase older adults’ willingness to use these interventions.

One potential explanation for this lower uptake is that older adults are less comfortable with emerging technologies. Indeed, research has shown that older adults who are more confident in their ability to use computers and the internet are more willing to adopt new technologies [48] and become long-term users of new digital programs [49]. Some researchers suggest that tablets may be the answer to increased adoption of technology among older adults [50]; however, our data suggest that only a small proportion of older adults on Happify used tablets to access the program. Generally, older adults still seemed to use smartphones, followed by computers, to access the program. Although more research is needed to explore the impact of optimizing digital interventions for tablets, these data suggest that the mode of delivery for digital interventions is not the root cause of lower uptake.

Rather, the issue may have more to do with a lack of familiarity with the interventions themselves than with technology. For example, qualitative research with adults aged ≥50 years suggests that the most common barrier to uptake of digital interventions in this age group is a lack of understanding [28]. More specifically, many participants were unaware of digital interventions, although they developed positive attitudes toward such interventions when introduced to them during the study session. However, other research suggests that awareness alone may not be sufficient to overcome older adults’ skepticism about how digital interventions can help improve their mental health [51]. Consequently, education to improve awareness among older adults may need to be coupled with support while learning new technologies to increase use [52].

### Designing Digital Interventions With Older Adults in Mind

Beyond efforts to introduce older adults into digital interventions, we also need to consider the unique needs of this population, which might make digital interventions designed for younger groups impractical for older adults. For instance, qualitative research with older adults suggests that although participants felt there were numerous benefits to technology, many reported concerns with usability based on age- or health-related changes in abilities (eg, difficulties with small screens and manual dexterity) [51,53]. Certain features, such as audio and voice recognition technology, may be required to increase the usability of digital interventions in this population [22]. Despite the proliferation of digital interventions available on the market, few, if any, have been developed specifically with older adults in mind. Given the increasing need for scalable mental health solutions among older adults, and the unique barriers to engaging with digital tools in this age group, product development that actively includes end user feedback will be imperative to the success of digital interventions among older adults, both in terms of uptake and efficacy. Indeed, other researchers have called for patient-centered or user-focused research with older adults as part of digital intervention development [54] or even exploring opportunities for participatory co-design [55]. Although qualitative research on older adults’ general perspectives on digital interventions and technology exists [28], more user-centered work on older adults as they engage with specific programs is needed.

### Strengths and Limitations

Although the strength of this study is its ability to provide insights into the real-world uptake of digital well-being interventions among older adults, it also has several limitations. First, given the lack of a control group, we could not determine whether the changes in well-being or anxiety symptoms were directly related to the intervention. We found that changes in outcomes were significantly different based on use, such that older adults who engaged with Happify at a minimal level showed less improvement in both well-being and anxiety symptoms compared with those who completed an average of at least two activities per week. This moderating effect of use suggests that the completion of Happify activities contributed, at least in part, to changes in outcomes. However, research with a control group is required to determine the causality. In particular, given recent criticisms that the effects of digital interventions are much weaker when compared to active controls [56], future research should include a rigorous control that would account for potential placebo effects as well as time.

Second, because of the naturalistic design, our analyses were limited to users who signed up for the program on their own. Consequently, it is likely that the older adults included in our analysis were not representative of all older adults. In particular, our sample predominantly consisted of older adults who identified as women. Although this is often the case in research on digital interventions and we found no significant effects of gender in either of our models, some research suggests that women may show greater improvements in mental health and well-being outcomes after engaging in digital interventions [57]. Consequently, these findings may not be generalizable to men.

This is compounded by the fact that our analysis included those users who engaged with the intervention for at least six weeks, who completed activities, and who completed at least two in-app assessments. Thus, our sample likely represents older adults who are early adopters of digital interventions and are more comfortable with the technology overall. Although it is important to understand how this group of older adults will respond to digital interventions, to determine whether these interventions are a viable solution to address the unmet need for mental health care among older adults, we need to test the usability of these interventions with a broader population of older adults. In particular, it is important to test the impact of digital interventions among older adults who may experience more barriers to engaging with these technologies, including those less familiar or comfortable with technology, those with conditions that might interfere with their ability to use digital tools (eg, cognitive deficits and mobility concerns), and those from diverse backgrounds.

Finally, although we were able to isolate users aged ≥65 years, because the question about age in the onboarding questionnaire is categorical, it is impossible to examine the continuous effects of age within this cohort. Research suggests that technology adoption may be even lower among adults aged ≥75 years [21] and that the benefits of digital interventions may be negatively related to age among older adults [23]. Conceivably, our effects may be driven by younger older adults, and in future research, it will be important to determine users’ age more precisely.

### Conclusions

As the population ages, the increasing need for mental health care coupled with the shortage of mental health professionals specializing in geriatric populations presents important concerns regarding unmet care needs. Although digital interventions have been presented as one way to address unmet needs in the general population, few studies have specifically examined the impact of such interventions on older adults. The current data add to the growing body of evidence suggesting that although older adults are less likely to begin using digital interventions without efforts to familiarize themselves with these interventions, those who engage with these interventions show corresponding improvements in their mental health. This suggests that digital interventions may present a viable opportunity to improve access to mental health care among older adults. Importantly, digital health programs may also help foster a sense of independence among older adults [58], offering them opportunities to address mental health concerns without feeling like they burden others [51]. Given the potential benefits, developing digital interventions specifically for older adults to address their unique needs and to provide education surrounding digital interventions to improve awareness of and comfort with these tools among older adults should be a priority.

## Abbreviations

CBT: cognitive behavioral therapy
GAD-2: Generalized Anxiety Disorder 2

## Data Availability

The data used for the analyses described in this paper are owned by Twill Inc and cannot be shared publicly without violating the terms, conditions and privacy policy agreed to by the users. Data may be shared upon request by contacting the corresponding authors.
